# Supplementary material for: Patients with Primary Central Nervous System Lymphoma Not Eligible for Clinical Trials: Prognostic Factors, Treatment and Outcome
Source: Cancers (Basel). 2021 Jun 11;13(12):2934. doi: 10.3390/cancers13122934 (PMC8230869; doi:10.3390/cancers13122934)
Supplement: Supplementary file 1 [file cancers-13-02934-s001.zip › cancers-1266139-supplementary.pdf]

cancers

MDPI

Supplementary Material

Patients with Primary Central Nervous System Lymphoma Not Eligible for Clinical Trials: Prognostic Factors, Treatment and Outcome

Sabine Seidel, Michelle Margold, Thomas Kowalski, Alexander Baraniskin, Roland Schroers, Agnieszka Korfel, Eckhard Thiel, Michael Weller, Peter Martus and Uwe Schlegel

Copyright: © 2021 by the authors. Licensee MDPI, Basel, Switzerland. This article is an open access article distributed under the terms and conditions of the Creative Commons Attribution (CC BY) license (http://creativecommons.org/licenses/by/4.0/).

Table S1. Chemotherapy protocol including rituxumab and temozolomide.

	Day 1	Day 2	Day 3	Day 4	Day 5	Day 6	Day 14
Cycle 1 (1 cycle = 28 days)							
Rituximab 375 mg/m^2 IV	x						x
Temozolomide 150 mg/m^2 PO		x	x	x	x	x	
Cycles 2–6[a] (1 cycle = 28 days)							
Rituximab 375 mg/m^2 IV	x						x
Temozolomide 200 mg/m^2 PO		x	x	x	x	x	

[a] Follow-up MRI planned after three cycles. Abbreviations: IV, intravenous; PO, per os.

Table S2. Outcome according to subgroup (n = 34).

Reason for Exclusion	No. of Patients	Age Range (y)	Response after Treatment	OS (months)
KPS < 30 because of PCNSL	6	66–81	CR n = 3<br>PD n = 1<br>Treatment discontinuation n = 2	1 (median)
KPS < 50[b] independent of diagnosis of PCNSL	1	60	PD	20
Psychiatric comorbidity	2	26/79	CR n = 2	141/2
Immunosuppression	7	52–75	CR/CRu n = 4<br>PD n = 2<br>TD n = 1	14 (median)
Previous malignancy	3	77–83	CR n = 2<br>PD n = 1	16 (median)
Heart insufficiency	3	66–74	CR n = 3	31

				(median)
Renal insufficiency	5	75–80	PR 1<br>PD 2<br>No treatment 2	2<br>(median)
KPS < 50[b] and psychiatric comorbidity	3	69–79	CR n = 1<br>PD n = 1<br>TD n = 1	2<br>(median)
KPS < 30 and heart insufficiency	1	74	CR	68
KPS < 30 and psychiatric comorbidity	1	62	CR	14
Heart and renal insufficiency	1	75	PD	14
Immunosuppression and renal insufficiency	1	80	No treatment	2

Abbreviations: CR, complete remission; CRu, complete remission unconfirmed; KPS, Karnofsky performance score; OS, overall survival; PD, progressive disease, PR, partial remission; TD, toxic death.

Table S3. Treatment characteristics (n = 34).

	No. of Patients
HDMTX application	
Yes	27 (79.4%)
No	7 (20.6%)
Rituximab application	
Yes	23 (67.6%)
No	11 (32.4%)
First-line treatment	
HDMTX and AraC-based polychemotherapy	10 (29.4%)
HDMTX and AraC-based polychemotherapy + ICV	13 (38.2%)
HDMTX and AraC-based polychemotherapy + ITH liposomal AraC	3 (8.8%)
Rituximab + temozolomide	3 (8.8%)
WBRT	1 (2.9%)
HD-ASCT	1 (2.9%)
Palliative treatment	3 (8.8%)
Second-line treatment (n = 12)	
Temozolomide	4
Rituximab + temozolomide	2
MTX + rituximab + temozolomide	1
WBRT	2
HD-ASCT	2
R-CHOP (systemic relapse)	1
Third-line treatment (n = 3)	
HD-ASCT	1
R-CHOP (systemic relapse)	1
Temsirolimus	1

Abbreviations: AraC, cytarabine; HDMTX, high-dose methotrexate; ICV, intracerebroventricular; ITH, intrathecal; WBRT, whole-brain radiation therapy; HD ASCT, high-dose chemotherapy with autologous stem-cell transplantation.
